# Controlled hyperventilation using a new ventilation navigator software: a feasibility study to provoke hypocapnia and respiratory alkalosis: A proposal for coronary artery vasoreactivity assessment

**DOI:** 10.1093/ehjimp/qyag070

**Published:** 2026-04-17

**Authors:** Attila Kardos, Qi Yong, Barbara Kardos, Nerea Sanfeliu Garces, Ellie Burgess, Kenneth Chan, Nikolaos Makris

**Affiliations:** Department of Cardiology, Translational Cardiovascular Research Group, Milton Keynes University Hospital NHS Foundation Trust, 8H Standing Way, Eaglestone, Milton Keynes MK6 5LD, UK; Faculty of Medicine and Health Sciences, University of Buckingham, Buckingham, UK; Faculty of Engineering and Applied Sciences, Cranfield University, Cranfield, UK; Department of Cardiology, Translational Cardiovascular Research Group, Milton Keynes University Hospital NHS Foundation Trust, 8H Standing Way, Eaglestone, Milton Keynes MK6 5LD, UK; Faculty of Medicine and Health Sciences, University of Buckingham, Buckingham, UK; Department of Cardiology, Translational Cardiovascular Research Group, Milton Keynes University Hospital NHS Foundation Trust, 8H Standing Way, Eaglestone, Milton Keynes MK6 5LD, UK; Faculty of Medicine and Health Sciences, University of Buckingham, Buckingham, UK; Department of Cardiology, Translational Cardiovascular Research Group, Milton Keynes University Hospital NHS Foundation Trust, 8H Standing Way, Eaglestone, Milton Keynes MK6 5LD, UK; Faculty of Medicine and Health Sciences, University of Buckingham, Buckingham, UK; Division of Cardiovascular Medicine, Acute Multidisciplinary Imaging & Interventional Centre, British Heart Foundation Centre of Research Excellence, Radcliffe Department of Medicine, University of Oxford, Oxford, UK; Department of Critical Care, Milton Keynes University Hospital NHS Foundation Trust, Milton Keynes, UK

**Keywords:** ventilation navigator, hyperventilation test, vasoreactivity, ANOCA

## Abstract

**Aims:**

To develop a ventilation navigator to provide controlled hyperventilation and to assess the feasibility to monitor hypocapnia and respiratory alkalosis in healthy subjects.

**Methods and results:**

We developed the Ventilation Navigator software using C++ and utilizing high-precision timers as an advanced platform for respiratory guidance and control, incorporating adjustable breathing rate, and tidal volume, a session timer for automatic cessation. Its graphical user interface is designed to deliver clear visual cues to the patient, facilitating controlled breathing. We assessed the utility of controlled hyperventilation in five healthy probands using end-tidal CO_2_ (ETCO_2_) against capillary CO_2_ and pH. We have successfully achieved respiratory alkalosis (pH >7.50) between 70% and 90% of the subjects’ baseline ETCO_2_. The linear mixed-effects modelling demonstrated a significant association between ETCO_2_ and capillary pH (*β* = −0.063, SE = 0.008, *P* < 0.001). There was a moderate, clinically relevant agreement between capillary and ETCO_2_ with an offset of 0.48 (1.50 – (−0.54)); however, ETCO_2_ was significantly associated with capillary CO_2_ (*β* = 0737, SE = 0.115, *P* < 0.001) after accounting for repeated measures within individuals. A strong within-individual associations for ETCO_2_ vs. capillary CO_2_ as demonstrated (*R*rm = 0.857, 95% confidence interval: 0.628–0.949, *P* < 0.001).

**Conclusion:**

We developed a ventilation navigator application to deliver a controlled hyperventilation and assess its feasibility to monitor hypocapnia and respiratory alkalosis in healthy subjects. We are proposing this controlled physiological test to be implemented in the routine diagnostic workup to investigate coronary vasoreactivity in patients having angina with non-occlusive coronary artery disease after it has been validated in patients.

## Lay Summary

In this study, we developed a respiration navigator software to govern patients breathing. We know that over-breathing can cause chest pain (angina) in patient with no blockages in their heart arteries because of the heart artery spasm. Our described controlled breathing technique, which we validated against capillary blood gas (blood CO_2_ and pH level) could provide a non-invasive tool to assess patients with angina and no blockages in their heart arteries.

## Graphical Abstract

**Graphical Abstract:**
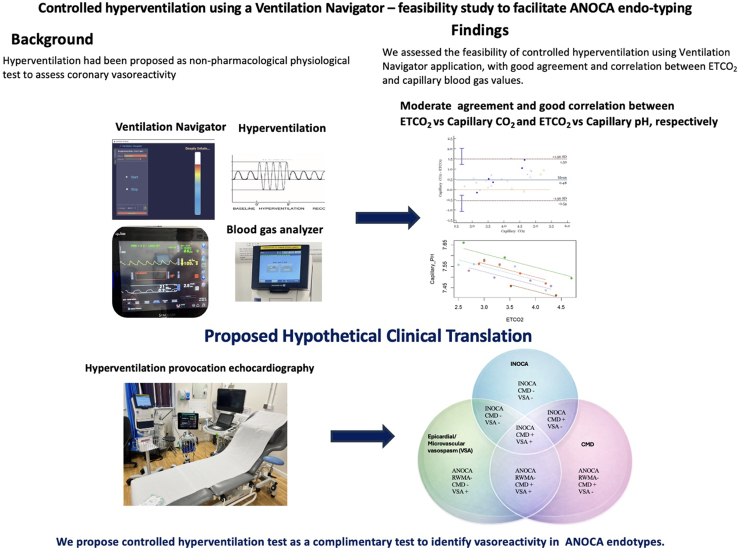
RWMA, regional wall motion abnormality; INOCA, ischaemia with non-occlusive coronary artery disease; ANOCA, angina with non-occlusive coronary artery disease; CFVR, coronary flow velocity reserve; VSA, vasospastic angina; CMD, coronary microvascular disease; MPSE, multi-parametric stress echocardiography; HPE, hyperventilation provocation echocardiography.

## Introduction

Chest pain is a common reason for primary care visit worldwide, accounting for about 1–2% of all consultations,^1–3^ approximately 5.6% of all emergency department visits in the USA annually^4^ and approximately 0.5% of outpatient clinic visits.^5^ Some of the patients will have occlusive coronary artery disease (CAD) to explain their symptoms; however, others who suffer from angina may have non-occlusive CAD (defined as no or <50% stenosis of the epicardial coronary arteries) termed as ANOCA. It is estimated that approximately 30–50% of males and 50–70% of females with chest pain have ANOCA. The pathophysiology has been widely investigated but far from fully established. It is believed that ANOCA is caused by coronary microvascular disease (CMD) and/or epicardial or microvascular coronary vasoreactivity, and often in combination.^6^

The current established methods, such as stress Positron Emitting Tomography (PET) perfusion images,^7,8^ stress perfusion cardiac magnetic resonance imaging (MRI),^9,10^ and stress echocardiography with Doppler coronary flow measurement, are the non-invasive ways to assess CMD based on myocardial or coronary flow reserve.^11,12^ However, ANOCA endotypes also include abnormal vasoreactivity of the epicardial or microvascular coronary circulation.^6,13,14^ These non-invasive tests cannot provide this information. Invasive functional coronary physiological testing (IFCA) is able to provide with the currently proposed endotypes for ANOCA patients to facilitate personalized management, although at a risk associated with invasive coronary angiography and wire manipulation in the coronary arteries during the test^15,16^ Albeit IFCA is not suitable for repeated testing to investigate therapeutic response in ANOCA patients.

Hyperventilation and cold pressor tests had been proposed as non-pharmacological and non-invasive physiological interventions to assess coronary vasoreactivity.^17,18^ Of these two tests, hyperventilation is deemed to be the more feasible, reproducible, and patient-friendly test. This causes respiratory alkalosis and sympathetic stimulation, which alters intracellular calcium handling and subsequent vasospasm as a mechanism.

In this paper, we are addressing the need for a precise, reproducible hyperventilation test. We developed a ventilation navigator application to deliver a controlled (tidal volume and respiratory rate modulated) hyperventilation and assessed its feasibility in healthy subjects during hypocapnia and related respiratory alkalosis, which in turn can be implemented to investigate coronary vasoreactivity in ANOCA patients once validated.

## Methods and protocol

### Ventilation navigator application

**Figure 1:**
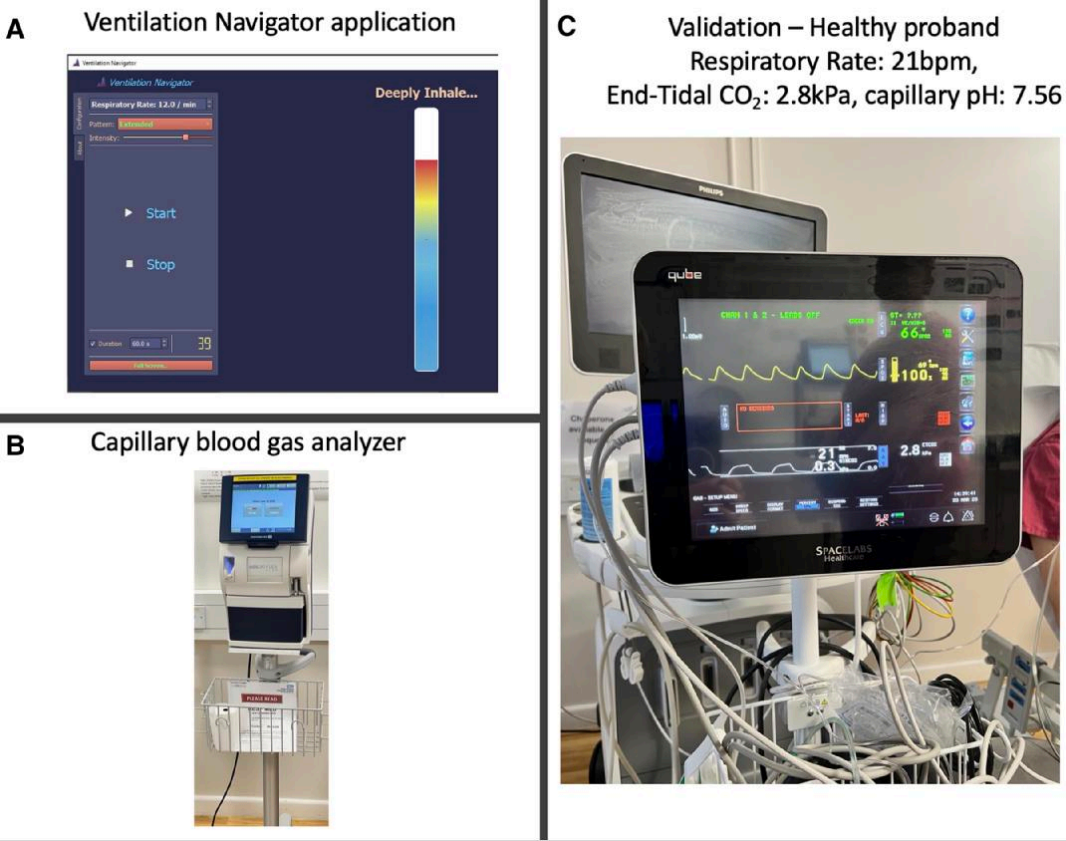
Feasibility of the ventilation navigator guided hyperventilation. Comparison of ETCO_2_ with capillary blood gas analysis. (*A*) Ventilation Navigator—to govern respiration rate, tidal volume (details in the Methods). (*B*) Blood gas monitor device for capillary pH, and CO_2_. (*C*) Hyperventilating healthy proband with the capnograph showing respiration rate of 21 breath/min that resulted an ETCO_2_ of 2.8 kPa corresponding with a capillary pH of 7.56 indicative of significant respiratory alkalosis necessary for provoking smooth muscle vasospasm of the coronary arteries if susceptible.

We have developed a software application that is able to govern patients’ ventilation frequency and tidal volume using a visual scale on a computer monitor (*Figure 1* and *Video 1*). The Ventilation Navigator software serves as an advanced platform for respiratory guidance and control, incorporating a comprehensive range of features such as adjustable breathing rate, and tidal volume, a session timer for automatic cessation, and customizable breathing pattern settings. Its graphical user interface is purposefully designed to deliver clear and engaging visual cues to the patient, facilitating precise regulation of respiratory movements whilst minimizing distractions and cognitive demand. Developed in C++ (C++23, New Jersey, USA) and utilizing high-precision timers, the software ensures optimal accuracy and reliability for clinical applications. The Ventilation Navigator can be used to adjust respiratory parameters to achieve controlled hyperventilation in turn to accomplish respiratory alkalosis sufficient to provoke vasospasm of the pre-arterioles/arterioles or capillaries level similar to the effect of acetylcholine intracoronary injection.

### Feasibility of the end-tidal CO_2_ (ETCO_2_) during controlled hyperventilation against capillary pH and CO_2_ in healthy subjects

We use ETCO_2_ monitoring via a Dual Nasal Cannula (3 m O_2_/CO_2_ Pk 25) as an indicator of hypocapnia as a gauge to assess the effect of controlled hyperventilation on blood pH. The ETCO_2_, representing the partial pressure of carbon dioxide at the end of exhalation (range 4.0–5.7 kPa as normocapnia). We validated the ETCO_2_ (using Qube Mini, 92516 Capnography Pod, Spacelab Healthcare, USA) against the capillary CO_2_ and capillary pH (Blood Gas analyser, ABL90 Flex Plus, UK) in five healthy volunteers (four female and one male age range 24–45 years). The healthy probands underwent controlled hyperventilation at different respiratory frequencies with fixed (non-quantified) tidal volume (by following the excursion [amplitude] of the up and down moving column on the visual scale during controlled breathing) for 3 min at each respiration rate to achieve the 90%, 80%, 70%, and 60% of their individual baseline ETCO_2_ calculated prior the test. The capillary blood gas was evaluated with a finger-prick from the non-dominant fingers at the end of each 3-min period for capillary CO_2_ and pH, whilst constantly monitoring the vital signs (heart rate, O_2_ saturation, ETCO_2_) and symptoms. The respiratory rates were adjusted on the Ventilation Navigator application on a PC for each volunteer, to visually follow the wide up-and-down moving column to navigate respiration. The effect of controlled hyperventilation on the capillary CO_2_ and capillary pH and ETCO_2_ in the individual healthy probands are listed in *Table 1*. The calibration related to the blood gas analyser has been provided in Supplementary data online, *Figure S1*.

**Table 1:** Controlled ventilation and changes in capillary blood gas and ETCO_2_ parameters in healthy probands

	Stages	Respiratory rate	ETCO_2_	Capillary CO_2_	Capillary pH	Symptoms
**Case 1 (f, 24)**						
	Base line ETCO_2_	8	4.2	4.25	7.439	No
	90%	15	3.6	2.65	7.55	No
	80%	17	3.3	3.15	7.554	No
	70%	21	2.8	3.13	7.56	No
	60%	26	2.5	3.05	7.557	Palmar numbness, headache
**Case 2 (f, 42)**						
	Base line ETCO_2_	8	4.7	4.63	7.495	No
	90%	9	3.4	3.5	7.591	Lightheaded
	80%	10	3	3.52	7.58	Lightheaded
	70%	10	2.6	2.76	7.66	Lightheaded
**Case 3 (m, 32)**						
	Base line ETCO_2_	17	4.2	4.83	7.471	No
	90%	8	3.7	4.11	7.517	No
	80%	10	3.3	3.66	7.559	No
	70%	19	2.9	3.61	7.562	No
	60%	25	3	3.44	7.575	No
**Case 4 (f, 36)**						
	Base line ETCO_2_	10	4.3	5.26	7.459	No
	90%	13	3.9	4.83	7.484	No
	80%	22	3.2	4.65	7.498	No
	70%	27	2.7	4.23	7.53	No
**Case 5 (f, 40)**						
	Base line ETCO_2_	22	4.4	5.16	7.415	No
	80%	14	3.52	4.57	7.459	No

Respiratory rate (breath/min), ETCO_2_ (kPa), capillary CO_2_ (kPa). M (): male (age), f (): female (age).

## Statistical analysis

Continuous variables were expressed as mean and 95% confidence intervals as all data were normally distributed (Shapiro–Wilk test). The parameters of the calculated ETCO_2_ subgroups (BL-90–80–70–60%) as a function of controlled hyperventilation were compared using Student’s *t*-test. The relationships between two variables (ETCO_2_ and capillary CO_2_ or capillary pH) were analysed using repeated measures of correlation (Rmc), and expressed as coefficients of repeated measurements (Rrm). The degree of agreement between capillary CO_2_ and ETCO_2_ were analysed with Bland–Altman method corrected to repeated measures for individual data points of the probands and as aggregate average for each proband. Statistical significance was defined as *P* < 0.05. MedCalc Version 23.4.5 (MedCalc Software Ltd, Mariakerke, Belgium) and R test R environment (R 4.5.3, The R Foundation for Statistical Computing, http://www.R-project.org) with Rmcorr package for repeated measures correlation and lme4 package for mixed linear model was used for analyses.

The study was carried out according to the principles of the Declaration of Helsinki and in compliance with Good Clinical Practice.

## Results

The calculated targeted ETCO_2_ derived from the individual baseline ETCO_2_ was closely achieved during controlled hyperventilation (calculated: 90–80–70% were achieved: 86–77–65% during hyperventilation). Each proband reached respiratory alkalosis defined as capillary pH ≥7.5. Three of the five probands reached pH ≥7.5 at the 90% of their baseline ETCO_2_ with minimal change in their respiratory rate (*Tables 1* and *2*). The difference in the measured variables during spontaneous breathing and with Ventilation Navigator—controlled breathing—was due to the controlled fixed tidal volume as a possible explanation. In one of the cases respiratory alkalosis was only achieved at the respiratory rate of 27 at the 70% value of the baseline ETCO_2_ (2.7 kPa). The case 5 had only two measurements at baseline and at 80% of her baseline ETCO_2_ due to the required calibration error of the blood gas analyser that resulted in termination of her test. Two of the subjects had symptoms related to respiratory alkalosis of mild headache and palmar numbness at 60% of baseline ETCO_2_ (2.5 kPa) and light-headedness at 90% of ETCO_2_ (3.4 kPa). The others had no symptoms.

**Table 2:** Summary table of controlled ventilation and changes in blood gas and ETCO_2_ parameters in healthy probands

	Stages (*n*)	Respiratory rate (bpm)	ETCO_2_ (kPa)	Capillary CO_2_ (kPa)	Capillary pH
**All**					
	Base line (5)	13 (5.2–20.8)	4.3 (4.1–4.6)	4.8 (4.3–5.3)	7.45 (7.42–7.49)
	90% (4)	11 (6.0–16.5)	3.7 (3.3–4.0)*	3.8 (2.3–5.2)*	7.54 (7.46–7.59)*
	80% (5)	15 (8.2–20.1)	3.3 (3.0–3.5)*#	3.9 (3.1–4.7)*	7.53 (7.47–7.59)*
	70% (4)	19 (8.0–30.4)	2.8 (2.5–3.0)*#$	3.4 (2.4_4.4)*	7.58 (7.49–7.67)*#
	60% (2)	26 (19.1–31.9)	2.8 (−0.43–5.9)	3.2 (0.8–5.7)*	7.57 (7.45–7.68)*

Values are mean (95% confidence interval). Note all data were normally distributed (Shapiro–Wilk test).

RR: *P* > 0.05 NS. Paired *t* test. ETCO_2_: *P* < 0.05 *BL, #90%, $80%. Capillary CO_2_: *P* < 0.05 *BL. Capillary pH: *P* < 0.05 *BL, #90%.

bpm, breath per minute; kPa, kilo Pascal.

### Relationship between ETCO_2_ vs. capillary pH and capillary CO_2_ vs. capillary pH

**Figure 2:**
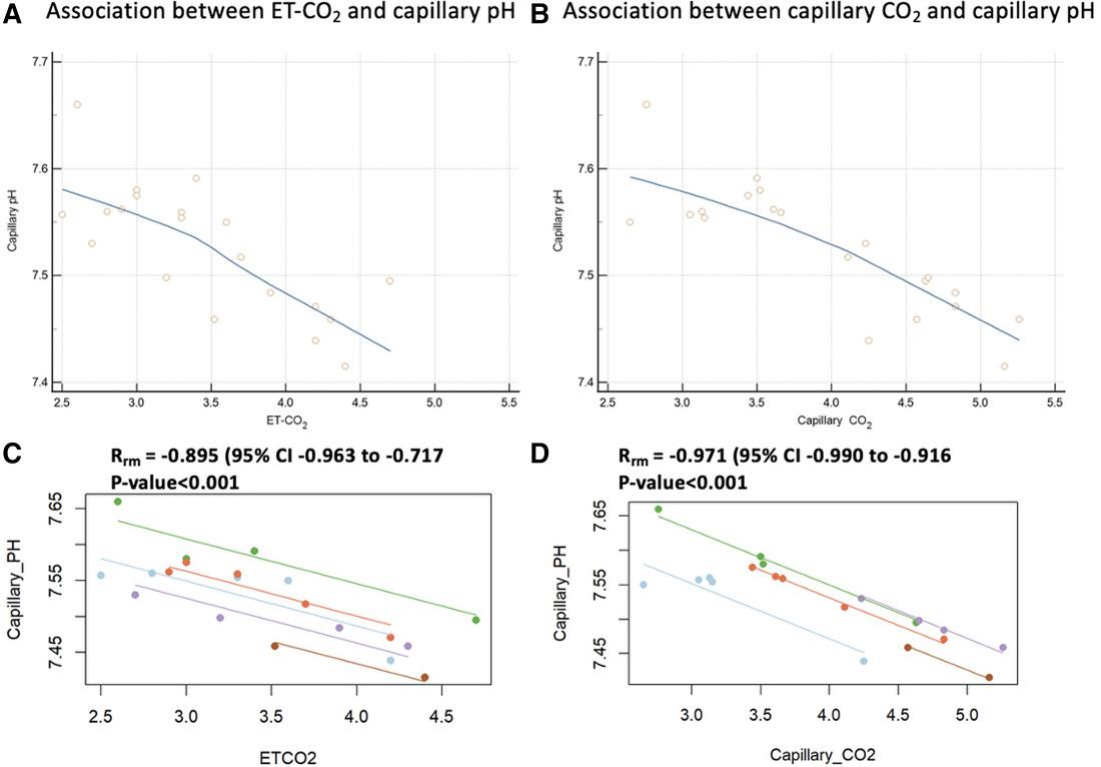
Relationship between ETCO_2_ vs. capillary pH and capillary CO_2_ vs. capillary pH. Responsiveness analysis shows good association between capillary pH and ET-CO_2_ or capillary CO_2_^−^ (*A*, *B*). Respiratory alkalosis defined as capillary pH >7.5 was achieved at different respiratory frequencies and corresponded to the ETCO_2_ of <3.5 kPa. Linear mixed-effects modelling between ETCO_2_ vs. capillary pH, (*C*) and Capillary CO_2_ vs. capillary pH (*D*). ETCO_2_ was significantly associated with capillary pH (*β* = −0.063, SE = 0.008, *P* < 0.001), after accounting for repeated measures within individuals. Note the high Rrm value and statistical significance as a sign of the strong within individual associations for both capillary pH vs. ETCO2 as well as capillary pH vs. capillary CO2. Regression lines between individual are mostly parallel with no outliers in slopes, suggesting good fit of the repeated measure correlation model.

Responsiveness analysis showed good association between capillary pH and ETCO_2_ or capillary CO_2_^−^ (*Figure 2*). Linear mixed-effects modelling demonstrated that ETCO_2_ was significantly associated with capillary pH after accounting for repeated measures within individuals (*β* = −0.063, SE = 0.008, *P* < 0.001). Significant association between capillary pH and capillary CO_2_ was also observed (*β* = −0.079, SE = 0.005, *P* < 0.001). Random slope models did not improve fit, supporting a common within-subject relationship. Similarly, for every 1-unit increase in capillary CO_2_, capillary pH decreased by approximately 0.079 units, after accounting for repeated measurements within subjects (*Figure 2*, Supplementary data online, *Figure S2*).

We found a strong within-individual associations for both capillary pH vs. ETCO_2_ as well as capillary pH vs. capillary CO_2_, as demonstrated by high Rrm value and statistical significance. Regression lines between individual are mostly parallel with no outliers in slopes, suggesting good fit of the repeated measure correlation model (*Figure 2*).

### Relationship between ETCO_2_ and capillary CO_2_

**Figure 3:**
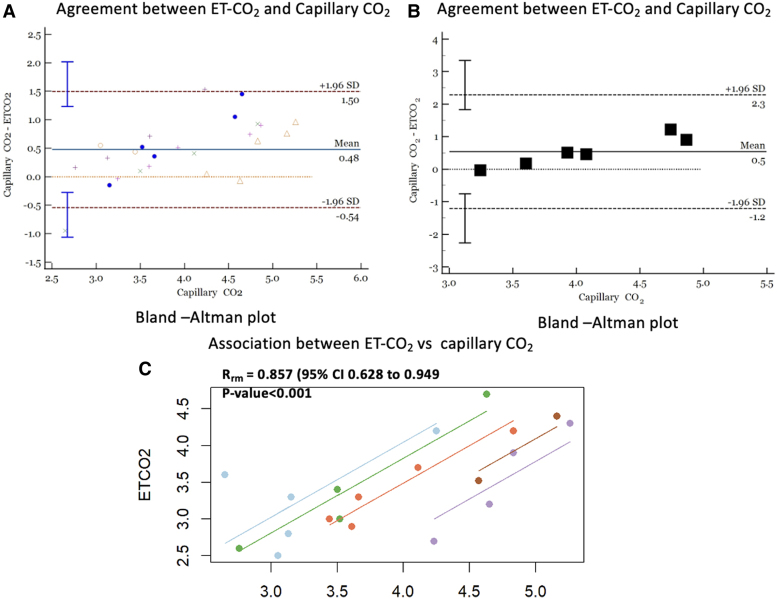
Relationship between ETCO_2_ and capillary CO_2_. Bland–Altman analysis corrected for repeat measurements analysis showed a moderate, clinically relevant agreement between capillary and ETCO_2_ with an offset of 0.48 (1.50 − (−0.54)) for individual data points of the probands (*A*) and aggregate average for each proband (*B*). Linear mixed-effects modelling between ETCO_2_ vs. capillary CO_2_. (*C*) ETCO_2_ was significantly associated with capillary CO_2_ (*β* = 0737, SE = 0.115, *P* < 0.001), after accounting for repeated measures within individuals strong within individual associations for both ETCO_2_ vs. capillary CO_2_ as demonstrated by high Rrm value and statistical significance. Regression lines between individual are mostly parallel with no outliers in slopes, suggesting good fit of the repeated measure correlation model.

Bland–Altman analysis corrected for repeat measurements analysis showed a moderate, clinically relevant agreement between capillary CO_2_ and ETCO_2_ with an offset of 0.48 (1.50 – (−0.54)) for individual data points of the probands and aggregate average for each proband (*Figure 3*).

Linear mixed-effects modelling between ETCO_2_ vs. capillary CO_2_ showed that ETCO_2_ was significantly associated with capillary CO_2_ (*β* = 0737, SE = 0.115, *P* < 0.001), after accounting for repeated measures within individuals. For every 1-unit increase in ETCO_2_ capillary CO_2_ increased by approximately 0.74 units, after accounting for repeated measurements within subjects. A strong within individual associations for both ETCO_2_ vs. capillary CO_2_ as demonstrated by high Rrm value and statistical significance. Regression lines between individual are mostly parallel with no outliers in slopes, suggesting good fit of the repeated measure correlation model (*Figure 3*). For every 1-unit increase in ETCO_2_, capillary CO_2_ increased by ∼0.74 units, after accounting for repeated measurements within subjects.

## Discussion

To be able to develop an entirely non-invasive, echocardiography-based pathway to help to determine ANOCA endotypes we have developed a Ventilation Navigator software to aid to deliver controlled hyperventilation. Hyperventilation provocation test is a well-established modality to elicit coronary vasospasm due to respiratory alkalosis and consequential calcium ion overload of the smooth muscle cells at the pre-arterioles, arterioles and capillary level.^17,18,19,20^ Using the controlled hyperventilation we found a moderate, clinically relevant agreement between ETCO_2_ and capillary CO_2_ with an offset of 0.48 kPa. Nevertheless, respiratory alkalosis, defined as blood pH ≥7.5, could be achieved between 90% and 70% of the baseline ETCO_2_ of individuals with the help of the Ventilation Navigator that is not dependent on excessive increase of the respiratory rate, once the tidal volume is standardized. Moreover, ETCO_2_ was significantly associated with capillary pH, which makes it an important marker for clinical monitoring of respiratory alkalosis during controlled hyperventilation.

### Proposed clinical translation of the findings

With our feasibility data, we would like to propose the implementation of the controlled hyperventilation-based echocardiography (HPE) to investigate patients with suspected ANOCA in the multi-parametric stress echocardiography (MPSE) protocol as a hypothetical pathway. Of course, our controlled hyperventilation protocol would need to be validated in prospective patients with suspected coronary vasoreactivity before widespread clinical implementation.

The controlled HPE can provide with a non-invasive assessment of epicardial and microvascular coronary vasoreactivity by monitoring patients’ symptoms, ST segments changes on 12-lead ECG (≥1 mm ST depression or elevation in 2 neighbouring leads), and left ventricular wall thickening using contrast-enhanced echocardiography (*Figure 3*) during hyperventilation-induced respiratory alkalosis. In addition to symptoms and ST changes on ECG, echocardiographic assessment of inducible ischaemia has improved the diagnostic accuracy from 60% to 91% during hyperventilation.^17^

Hyperventilation–provocation echocardiography showed a specificity of 100%, a sensitivity of 84%, a positive predictive value of 100%, and a negative predictive value of 92% in a patient with chest pain.^21^ Hyperventilation test has a Class IIa recommendation by the Japanese Society of Cardiology guidelines for diagnosing VSA.^18^

**Figure 4:**
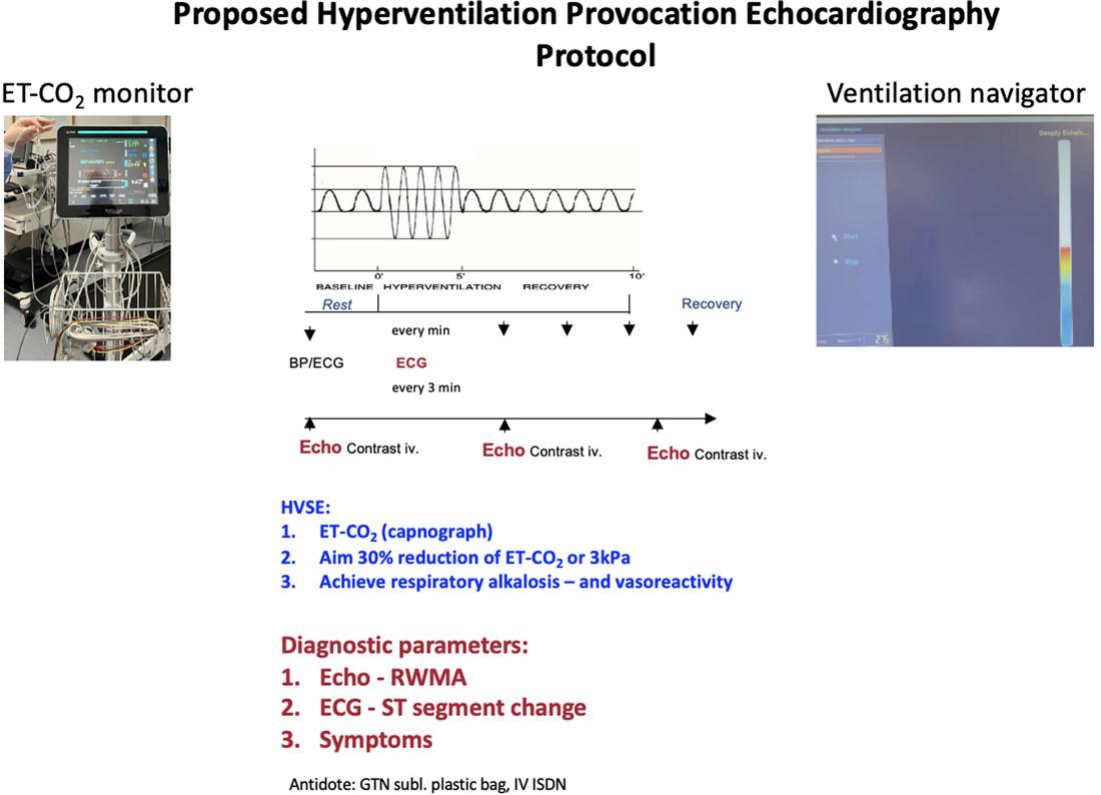
Hypothetical hyperventilation provocation echocardiography protocol guided by the ventilation navigator application and controlled by ETCO_2_. RWMA, regional wall motion abnormality; ECG, electrocardiogram.

Since its diagnostic accuracy, HPE combined with MPSE, as a fully non-invasive pathway, can also be used as a triaging tool to select patients who need further testing by IFCA. *Figure 4* offers a conceptual overview of the diagnostic pathway for suspected ANOCA, based on MPSE to assess CMD and HPE to address vasoreactivity and the relevant endotypes to guide personalized management. ANOCA patients with no inducible ischaemia with normal CFVR and negative vasoreactivity testing should be considered for IFCA as a confirmatory investigation especially if symptoms and cardiovascular risk factors for ANOCA are convincing.

### Pathophysiology of ANOCA

Disease of the coronary macro- and microcirculation can develop due to structural or functional changes. The structural abnormality entails diffuse or focal atherosclerosis, intimal thickening, smooth muscle cell thickening or proliferation, perivascular fibrosis/inflammation, capillary obstruction or reduced capillary diameter or density (aka rarefaction). Functional anomalies can be abnormal vasoconstriction, impaired vasodilatation, endothelial dysfunction.^22^ Twenty-five percent of patients with ANOCA had inducible ischaemia, 50% have CMD because of abnormal endothelium independent vasodilatation, 60% have epicardial or microvascular vasoreactivity (6).

### Diagnostic modalities in assessing ANOCA

To be able to address appropriate therapy in such patients, the mechanism must be identified, however challenging it is. Based on IFCA, three different endotypes of ANOCA have been described: CMD, epicardial vasospastic angina, and microvascular vasospastic angina. Beyond the three main endotypes, there are four different overlap endotypes as a combination of two or all three of the individual endotypes.^22^ Non-invasive functional tests have developed to assess indirectly the effect of the structural and functional abnormalities of the coronary microcirculation, which has IIa and IIb recommendation by the current guidelines to assess suspected ANOCA patients.^6,23^ PET, Cardiac MRI are assessing in a semi-quantitative and quantitative way the myocardial or coronary flow reserve (CFR/MBFR) during maximal vasodilatation as a ratio of the maximal over the resting flow. Doppler echocardiography-based LAD coronary flow velocity reserve (CFVR) during stress echocardiogram has been shown to have both diagnostic and prognostic value.^12^ As their diagnostic utility neither of the non-invasive tests can provide with the assessment of macro or microvascular vasoreactivity. In addition, IFCA for the evaluation of vasoreactivity uses ‘pharmacological dose’ of intracoronary acetylcholine raising queries over its safety and diagnostic value.^24^ To overcome the limitations of non-invasive modalities to provide comprehensive classification of ANOCA endotypes and to avoid IFCA tests with its inherent complications and cost implications we identified the need for a physiological, validated safe test to be implemented for vasoreactivity testing that can complement Doppler echocardiography derived CFVR to diagnose CMD to identify ANOCA endotypes non-invasively.

## Limitation

Our study has several limitations. First we used only five healthy proband to assess feasibility of controlled hyperventilation induced hypocapnia and respiratory alkalosis. We have exposed these subjects to different respiratory rate to achieve their 90–80–70–60% baseline ETCO_2_ and to calibrate it against the capillary blood gas values, thus we had 20 comparative points to analyse. One may argue that patients especially with known respiratory disease the respiratory alkalosis to investigate vasoreactivity would be at different ETCO_2_ level. Second, we validated ETCO_2_ against capillary CO_2_ and pH rather than arterial blood gas (ABG). While capillary sampling is practical, it is not equivalent to arterial measurement, especially under dynamic respiratory manipulation. However, this is more relevant for measures of oxygenation than for pH and PaCO_2_. There is good evidence that capillary blood gas provides good concordance with these ABG parameters, with capillary pH being found to accurately reflect arterial over a wide range of pH.^25^ We felt that the risks of invasive arterial puncture or cannulation of our healthy probands outweighed the benefits of ‘gold-standard’ measurement, particularly as oxygenation was not relevant to this study. Furthermore, since in a dynamic system, changes in capillary pH must necessarily *follow* changes in arterial pH, the use of capillary pH is highly specific for the presence of an alkalosis.

Controlled hyperventilation with the Ventilation Navigator system can add an extra control for the accurate assessment of macro/micro-vascular vasoactivity, nevertheless, it is unable to differentiate between epicardial or microvascular spasm. The 91% negative predictive value of HPE would not make it possible in some patients with otherwise typical angina to rule out vasospastic angina. Patients with either non-conclusive MPSE or HPE should be considered for further testing by IFCA to complete the diagnostic workup. Alternative physiological test such as cold-pressor test would provide similar vasospastic stimulus; however, its execution is more difficult and less tolerated by patients.^19,20^

Once the HPE has been validated exclusion criteria would be defined and provided, for patients in whom hyperventilation can adversely affect their underlying medical conditions and could put patients at risk of serious adverse reaction. Patients in whom hyperventilation provocation would cause safety concerns are those with severe pulmonary disease (such as severe asthma, chronic obstructive pulmonary disease, interstitial lung disease), recent cerebrovascular disease (TIA, stroke, intracranial haemorrhage), or known cerebral artery aneurysm, or sustained ventricular arrythmia.

## Conclusion

We assessed the feasibility of controlled hyperventilation using Ventilation Navigator application, with good agreement and correlation between ETCO_2_ and capillary blood gas values. We found that by monitoring ETCO_2_ and controlling respiration rate, respiratory alkalosis can safely be reached as a provocation test for vasoreactivity. We propose controlled hyperventilation provocation test as a complimentary test to identify vasoreactivity forms of ANOCA endotypes. MPSE in combination with HPE can diagnose CMD and endothelium dependent coronary vasospasm in ANOCA patients. The seven distinct endotypes of ANOCA can be identified to deliver targeted therapy for symptomatic control, to avoid recurrent hospital admissions, monitor therapy and reduce healthcare costs. Of course prior clinical implementation this pathway will have to be proven valid in ANOCA/INOCA patients (*Graphical Abstract*).

## Supplementary Material

qyag070_Supplementary_Data

## Data Availability

Data used in this study can be available from the corresponding author at reasonable request.

## Lead author biography

Professor Attila Kardos is a consultant cardiologist in Milton Keynes University Hospital, where he is also the Associate Director of Research and the Director of the Translational Cardiovascular Research Group that is affiliated to University of Buckingham. He has a Honorary Chair at the University of Buckingham as Professor of Cardiovascular Medicine, and holds a Visiting Professorial position at both Cranfield and Open Universities. He is an Honorary Senior Lecturer to the Division of Cardiovascular Medicine at the Radcliffe Department of Medicine, University of Oxford. He is the director of the Multimodality Cardiovascular Imaging Services in Milton Keynes. He has over 25 years of research experience and 32 years of clinical experience of which the last 23 years as a consultant.Professor Kardos is a chief investigator in several research trials and projects. His research interest expands to cardiovascular imaging modalities (CCTA and stress echocardiography) pathophysiology of stable and acute coronary syndromes in particular ANOCA, AI algorithm in cardiovascular diagnostics, and predictive modelling, AI and Robotics in cardiovascular imaging. He has published over 125 peer-reviewed journal articles, H-index: 24; He has been a Invited as well as Keynote speaker at National and International Conferences >120. He has supervised 5 MSc, DPhil/PhD/MD students Professor Kardos is a Fellow of the RCP and the European Society of Cardiology and a member of several International Societies such as EACVI, SCCT, BCS, AHA, ACC, ASE etc.). Associate Editor of Frontiers in Cardiovascular Medicine, Journal of Clinical Medicine, Cardiovascular Endocrinology & Metabolism, and reviewer to several Journals e.g. The Lancet, Open Heart, Open BMJ, JMIR, Frontiers in Cardiovascular Medicine.
