# Supplementary material for: Controlled hyperventilation using a new ventilation navigator software: a feasibility study to provoke hypocapnia and respiratory alkalosis: A proposal for coronary artery vasoreactivity assessment
Source: Eur Heart J Imaging Methods Pract. 2026 Apr 17;4(1):qyag070. doi: 10.1093/ehjimp/qyag070 (PMC13270313; doi:10.1093/ehjimp/qyag070)
Supplement: qyag070_Supplementary_Data [file qyag070_Supplementary_Data.docx]

Supplementary Document

**Controlled Hyperventilation Using a New Ventilation Navigator Software – a Feasibility Study to Provoke Hypocapnia and Respiratory Alkalosis**

**A proposal for coronary artery vasoreactivity assessment**

Attila Kardos MD., PhD., FRCP. FESC ^1,2^ Qi Yong MSc ^3^, Barbara Kardos BSc^1,2^, Nerea Sanfeliu Garces BSc^1,2^ , Ellie Burgess BSc^1,2^, Kenneth Chan, MRCP^4,^ Nikolaos Makris MB BChir MRCP, FRCA FFICM ^5^.

**Affiliations:**

1 Department of Cardiology, Translational Cardiovascular Research Group, Milton Keynes University Hospital NHS Foundation Trust, Milton Keynes, United Kingdom

2 Faculty of Medicine and Health Sciences, University of Buckingham, Buckingham, United Kingdom

3 Faculty of Engineering and Applied Sciences, Cranfield University, Cranfield, United Kingdom

4 Acute Multidisciplinary Imaging & Interventional Centre, British Heart Foundation

Centre of Research Excellence, Division of Cardiovascular Medicine, Radcliffe Department of Medicine university of Oxford, Oxford, UK

5 Department of Critical Care, Milton Keynes University Hospital NHS Foundation Trust, Milton Keynes, United Kingdom

**Corresponding Author:**

Prof. Attila Kardos, MD, FRCP, PhD, FESC,

Department of Cardiology, Translational Cardiovascular Research Group, Milton Keynes University Hospital NHS Foundation Trust, Milton Keynes, United Kingdom, 8H Standing Way, Eaglestone, Milton Keynes, MK6 5LD UK.

Email: [attila.kardos@cardiov.ox.ac.uk](mailto:attila.kardos@cardiov.ox.ac.uk)

**Calibration of the blood gas analyser during the study period in March 2025.**

The analyser is programmed to run a calibration every 24hrs, when consumables are replaced or if it detects a sample error that may impact future results (i.e. a blood clot in the sample). The calibration involves checking the linearity across two control levels, as well as measuring the sensitivity and stability. The analyser is also programmed to run an internal quality control (IQC) level every 8 hours or whenever consumables are changed. The results are checked to be within the manufacturer's ranges and reviewed against Westgard rules. If a parameter fails a calibration or IQC it is deactivated for patient testing. An alert is sent to the quality control team, who will troubleshoot the issue.


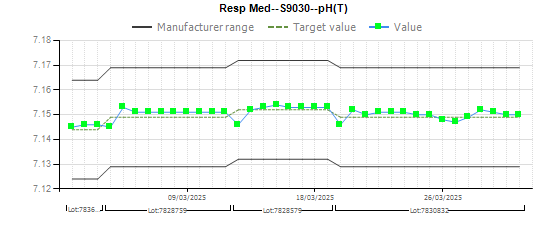


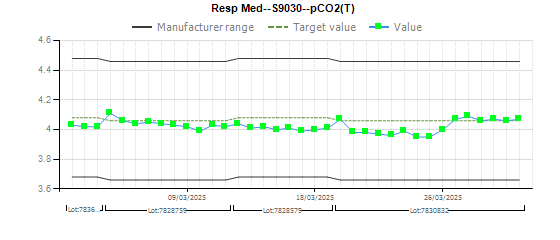


**Supplementary Figure 1.**

These graphs show the Levey-Jennings plots for the selected analysers for pH and CO_2_ parameters during a period of time. It shows the lot number and lot change, the measured value, the control and/or manufacturer range and the target value. The report helps compare parameters across levels.

The Calibration report is attached as a spreadsheet for consideration (to the discretion of the Editor)

**Supplementary Figure 2**. - Statistical analysis - Linear mixed model:


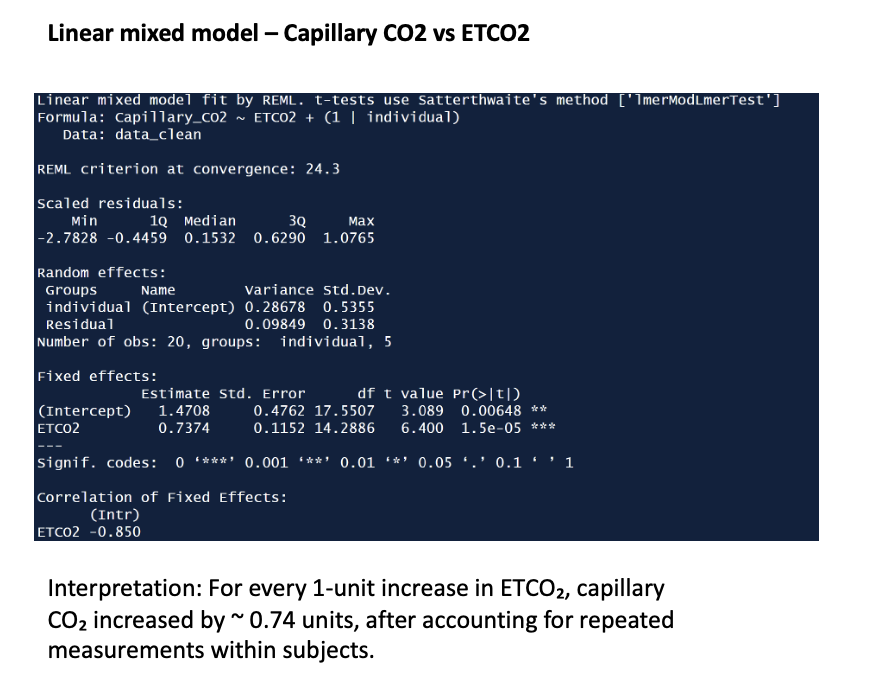


**Linear mixed model – ETCO_2_ vs Capillary pH and vs Capillary CO_2_ vs Capillary pH**


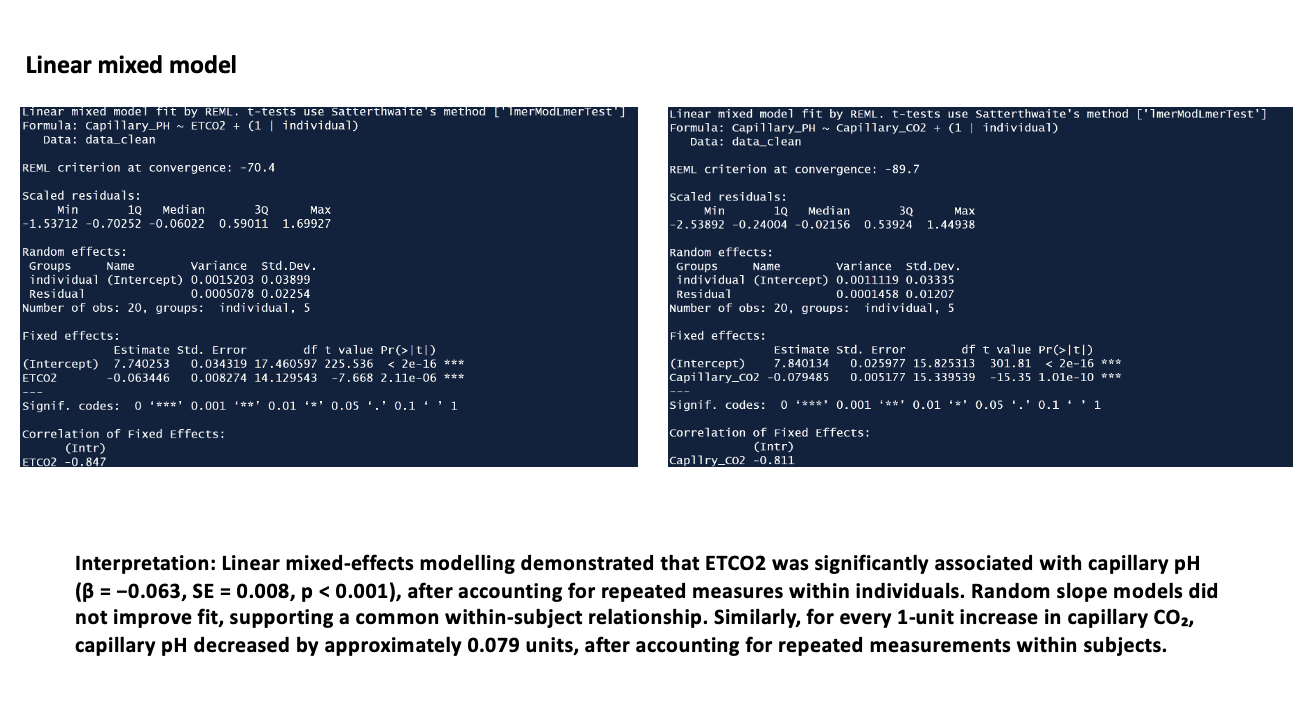


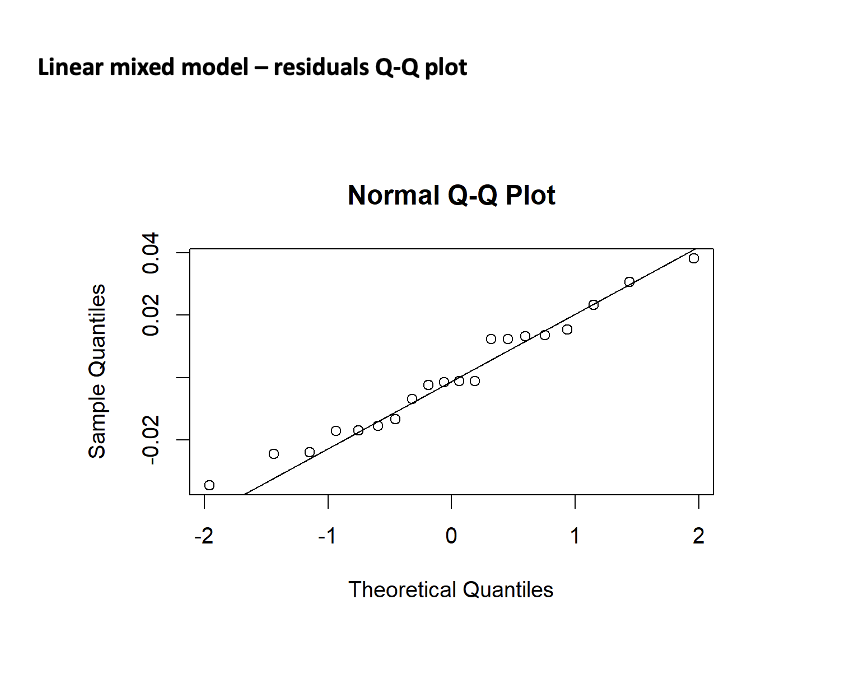


**Reference**:

Bakdash JZ, Marusich LR. Repeated Measures Correlation. Front Psychol. 2017 Apr 7;8:456. doi: 10.3389/fpsyg.2017.00456. Erratum in: Front Psychol. 2019 May 28;10:1201. doi: 10.3389/fpsyg.2019.01201. PMID: 28439244; PMCID: PMC5383908.
